# Vaccination with an HIV T-cell immunogen induces alterations in the mouse gut microbiota

**DOI:** 10.1038/s41522-022-00368-y

**Published:** 2022-12-30

**Authors:** Alessandra Borgognone, Aleix Elizalde-Torrent, Maria Casadellà, Luis Romero, Tuixent Escribà, Mariona Parera, Francesc Català-Moll, Marc Noguera-Julian, Christian Brander, Alex Olvera, Roger Paredes

**Affiliations:** 1 IrsiCaixa AIDS Research Institute, Badalona, Spain; 2 Universitat Autònoma de Barcelona (UAB), Barcelona, Catalonia Spain; 3 Universitat de Vic-Universitat Central de Catalunya (UVic-UCC), Vic, Spain; 4 Ciber of Infectious Diseases CIBERINFEC, ISCIII, Madrid, Spain; 5 Institució Catalana de Recerca i Estudis Avançats (ICREA), Barcelona, Spain; 6 AELIX Therapeutics, Barcelona, Spain; 7 Center for Global Health and Diseases, Department of Pathology, Case Western Reserve University, Cleveland, OH USA; 8 Fundació Lluita contra les Infeccions, Hospital Universitari Germans Trias i Pujol, Badalona, Catalonia Spain; 9 Department of Infectious Diseases, Hospital Universitari Germans Trias i Pujol, Badalona, Catalonia Spain

**Keywords:** Microbiome, Next-generation sequencing

## Abstract

The gut microbiota is emerging as a crucial factor modulating vaccine responses; however, few studies have investigated if vaccines, in turn, can alter the microbiota and to what extent such changes may improve vaccine efficacy. To understand the effect of T-cell vaccination on the gut microbiome, we administered an HIV-1 T-cell immunogen (HTI arm) or PBS (control, Mock arm) to C57Bl/6 mice following a heterologous prime-boost scheme. The longitudinal dynamics of the mice gut microbiota was characterized by 16 S ribosomal RNA sequencing in fecal samples collected from cages, as well as from three gut sections (cecum, small and large intestine). Serum and spleen cells were obtained at the last time point of the study to assess immune correlates using IFNγ ELISPOT and cytokine Luminex^®^ assays. Compared with Mock, HTI-vaccinated mice were enriched in *Clostridiales* genera (*Eubacterium xylanophilum* group, *Roseburia* and *Ruminococcus*) known as primary contributors of anti-inflammatory metabolites, such as short-chain fatty acids. Such shift was observed after the first HTI dose and remained throughout the study follow-up (18 weeks). However, the enriched *Clostridiales* genera were different between feces and gut sections. The abundance of bacteria enriched in vaccinated animals positively correlated with HTI-specific T-cell responses and a set of pro-inflammatory cytokines, such as IL-6. This longitudinal analysis indicates that, in mice, T-cell vaccination may promote an increase in gut bacteria known to produce anti-inflammatory molecules, which in turn correlate with proinflammatory cytokines, suggesting an adaptation of the gut microbial milieu to T-cell-induced systemic inflammation.

## Introduction

It has been well established that the resident gastrointestinal microbiota plays a pivotal role in the development and regulation of both innate and adaptive immune responses^1^. Of the different determinants of the adaptive immune response, T cell response polarization might be especially affected by the gut bacteria composition^2^. In fact, the absence of specific bacteria has been associated with reduced frequencies of Treg cells in the gut^3^ or dysregulated Th17 cell responses in germ-free and antibiotic-treated mice^4^. Abnormal ratios of these T cell subpopulations (Th17 and Treg) have been frequently associated with metabolic or immunologic diseases^5^.

Given the tight interaction and co-evolution of gut microbiota and immune system^6^, increasing evidence suggests that certain bacterial taxa might also modulate vaccine-induced immune responses^7^, by exerting local or systemic effects^8^. In recent years, several clinical and animal studies have shown that the composition and functions of the gut microbiota are crucial determinants of the immunogenicity and efficacy of oral and injectable vaccines^9,10^. So far, most work in this area has been focused on the role of the microbiota in modulating vaccine-induced antibody responses^7^. Of note, the importance of T cell-mediated immunity has been increasingly recognized in the protection induced by several immunization strategies, including those against pathogens such as HIV-1^11^ or SARS-CoV-2^12^. Yet, a limited number of studies have directly investigated the relationship between the pre-existing gut microbiota and T cell responses to vaccination^13^.

The effect of vaccine administration on the intestinal microbiota has gathered even less attention. Previous works investigating the effects of HIV-1^14,15^ and oral typhoid^16^ vaccines on the human gut microbiota reported no discernible perturbations of the microbial community structure induced by vaccination. In contrast, an increase in the *Firmicutes*/*Bacteroidetes* ratio following HIV-1 DNA/protein immunization was observed in the rectal microbiome of non-human primates^17^. Also, a recent study comparing two SARS-CoV-2 vaccination strategies reported changes in the human gut microbiota 1 month after immunization, characterized by lower alpha diversity and increased abundance of a certain bacterial species, such as *Bacteroides caccae*^18^. Collectively this provides at least circumstantial evidence that vaccination can influence the resident microbiota. Therefore, understanding how vaccines promote changes in the host intestinal immune milieu and alter the microbial community structure, may provide important insight into this interplay and, in turn, the identification of specific bacteria that positively modulate vaccination outcome.

In the present study, HIV-1 T-cell immunogen (HTI) expressing vectors^19,20^ were used to assess vaccine-induced changes in the gut microbiota and to identify potential correlates with the immunological response to vaccination in a murine model. The HTI immunogenicity has been tested in both mice^21,22^ and humans^23^ using different vaccine vectors (such as DNA, Chimpanzee Adenovirus, ChAdOx1; Modified Vaccine virus Ankara, MVA and Bacillus Calmette–Guérin, BCG). Based on this previous evidence, mouse strain C57Bl/6 and a three DNA prime followed by a ChAdOx.1—MVA boost were selected to achieve maximal immunogenicity. 16 S rRNA gene sequencing was used to characterize longitudinal fecal microbiome and intestinal content profiles in mice receiving a prime-boost HTI immunization regimen or PBS (control group). Correlations between gut microbial signatures, T cell vaccine response and cytokines after full immunization were also assessed.

## Results

### Study design and sequencing analysis

**Fig. 1:**
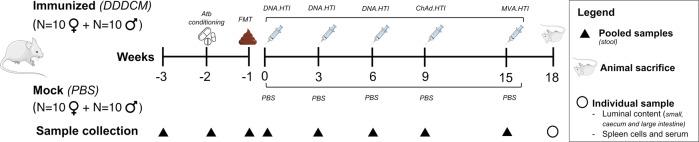
Study design and sample collection. Female and male mice were immunized with an HIV T-cell vaccine (three DNA.HTI primes followed by a ChAd.HTI and MVA.HTI booster; *n* = 20) or a mock vaccine (PBS; *n* = 20). Antibiotic conditioning followed by fecal microbiota transfer was performed prior to vaccination to homogenize the intestinal content across all animals. Collection of samples, including stool, luminal content, serum and spleen was completed at the indicated time points. Abbreviations: FMT fecal microbiota transfer, Atb Antibiotic, PBS phosphate-buffered saline. The Figure was partly generated using Servier Medical Art, provided by Servier, licensed under a Creative Commons Attribution 3.0 unported license.

To investigate the effects of HTI vaccination on the gut microbiota, two experimental groups receiving the HTI immunogen (Immunized, *n* = 20) or PBS (Mock, *n* = 20) were compared (Fig. 1). Fecal samples were collected longitudinally from cages and from three gut sections for gut microbiome profiling; serum and splenocytes were obtained at the end of the study to assess vaccine immunogenicity and cytokine profiles, respectively (Supplementary Fig. 1). Two weeks before vaccination, the mice gut microbiota was homogenized by antibiotic conditioning followed by mouse-to-mouse fecal microbiota transfer (FMT) to reduce baseline variability, as detailed in Supplementary Text and Supplementary Fig. 2. Baseline mouse characteristics at animal arrival (-3w; Supplementary Tables 1 and 2) and pre-vaccination (0w; Table 1 and Supplementary Table 3) are reported.

**Table 1:** Baseline characteristics at baseline (0 week, pre-vaccination).

Group	Mouse Line	Age (weeks)	Cage	N mice	Sex	Weight (g ± SD)1^1^	Sequencing Batch	Microbiota composition^2^
Mock	C57BL/6JOlaHsd	9	C19	5	F	19.3 ± 0.9	B1	*Lachnospiraceae NK4A136 group* (10.8%), *Bifidobacterium* (5.7%), *Bacteroides* (5.3%), *Lactobacillus* (3.4%), *Turicibacter* (1.7%)
Mock	C57BL/6JOlaHsd	9	C20	5	M	25.4 ± 1.4	B1	*Bacteroides* (11.4%), *Lachnospiraceae NK4A136 group* (7.9%), *Bifidobacterium* (6.2%), *Alistipes* (2.5%), *Anaerostipes* (1.7%)
Mock	C57BL/6JOlaHsd	9	C31	5	F	19.4 ± 1	B2	*Bacteroides* (16.3%), *Lachnospiraceae NK4A136 group* (12.2%), *Bifidobacterium* (5.1%), *Lactobacillus* (3.1%), *Mucispirillum* (2.7%)
Mock	C57BL/6JOlaHsd	9	C32	5	M	25.3 ± 1.3	B2	*Bacteroides* (19.2%), *Bifidobacterium* (10.4%), *Lachnospiraceae NK4A136 group* (10.1%), *Alistipes* (3.2%), *Lactobacillus* (2.1%)
Immunized	C57BL/6JOlaHsd	9	C21	5	F	19.5 ± 0.9	B1	*Bacteroides* (11.7%), *Lachnospiraceae NK4A136 group* (11.7%), *Bifidobacterium* (6.6%), *Alistipes* (1.8%), *Lactobacillus* (3.9%)
Immunized	C57BL/6JOlaHsd	9	C22	5	M	25.6 ± 2	B1	*Lachnospiraceae NK4A136 group* (14.1%), *Bifidobacterium* (7.4%), *Bacteroides* (6.7%), *Alistipes* (3.2%), *Lactobacillus* (1.6%),
Immunized	C57BL/6JOlaHsd	9	C29	5	F	17.2 ± 0.6	B2	*Bacteroides* (14.1%), *Bifidobacterium* (12.9%), *Lachnospiraceae NK4A136 group* (8.5%), *Lactobacillus (3.4%), Faecalibaculum* (2.1%)

^1^mean grams and sd.

^2^percentage of top 5 most abundant taxa; complete list of taxa is provided in Supplementary Table 3.

Mock and Immunized longitudinal samples had no differences in the number of high-quality filtered reads, with a median of 52,092 and 46,584 reads, respectively (Supplementary Fig. 3a and Table S4). Negative extraction (*n* = 12) and no-template PCR (*n* = 11) controls had significantly lower number of reads (5966 and 1444 mean read number, respectively) compared to the experimental groups (Supplementary Fig. 3a). Despite the sequencing depth was higher in batch 1 (Supplementary Fig. 3b), no substantial impact on data structure was observed after assessing for differences between the two batches (Supplementary Fig. 4) (batch was included as a covariate in testing for pooled fecal samples collected from cages).

### Longitudinal assessment of microbiota composition and diversity after HTI vaccination

Prior vaccination, samples displayed similar bacterial composition (0 week, Supplementary Fig. 2) and only the genus *A2* was increased in the Mock group (Supplementary Fig. 5a). After segregating by sex, female mice showed higher abundance of *Lactobacillus* and *Muribaculum* before vaccination (Supplementary Fig. 5b).

**Fig. 2:**
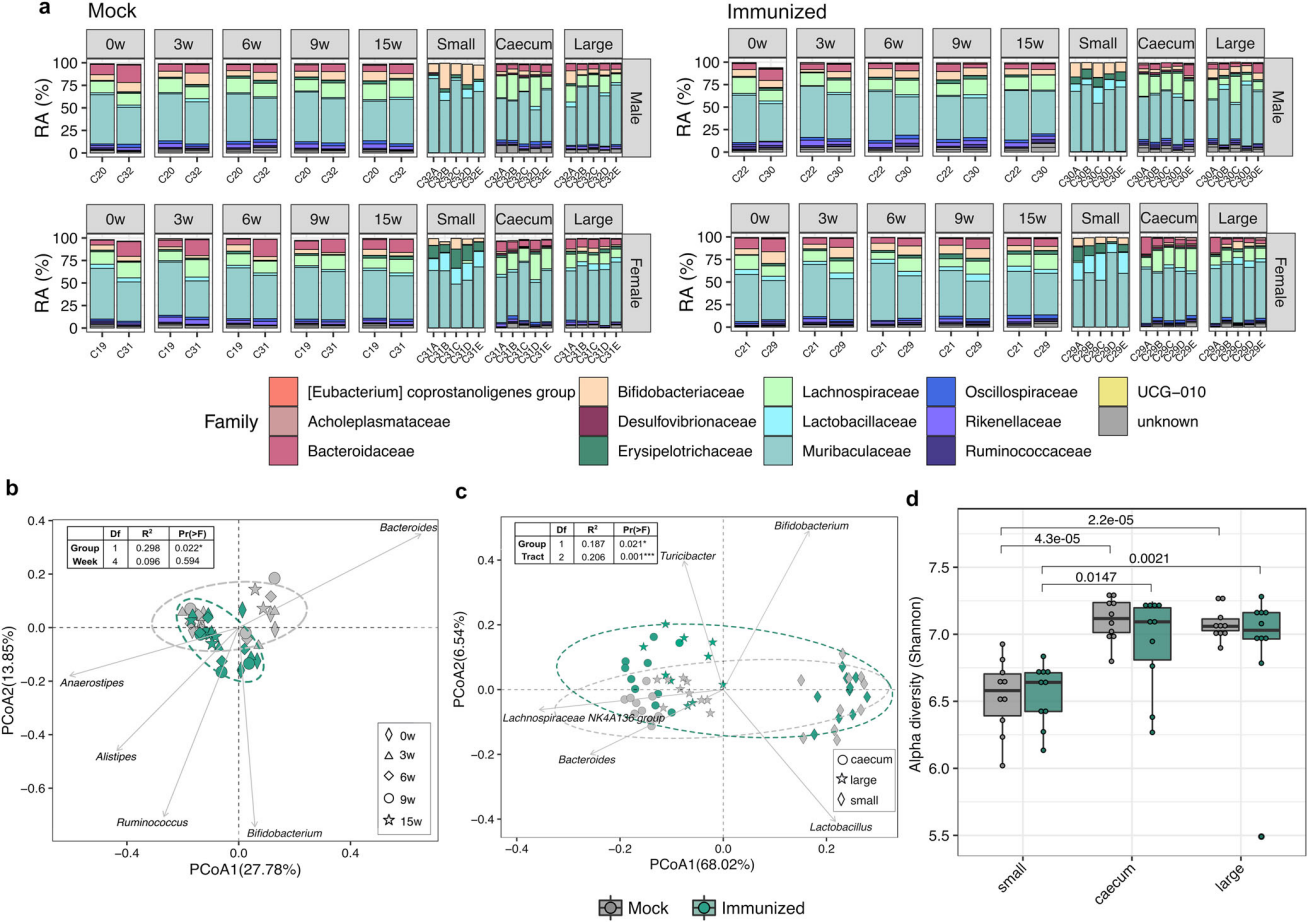
Temporal dynamics in microbiome composition after vaccination. **a** Stacked bar chart displaying the relative abundance of bacterial families (top 15 most abundant) at different timepoints in HTI-vaccinated (Immunized) or PBS-vaccinated (Mock) mice. CageID (pooled feces) and individual samples for each cage (intestinal content) are reported in the *x*-axis. PCoA biplot based on the first and second components (Bray–Curtis distances at genus level) of (**b**) fecal samples from cages (**c**) and from gut sections (small intestine, caecum and large intestine). Biplots report genera with the top 5 effects on the community composition, with vector position indicating the direction of the effect. Arbitrary ellipses were drawn to facilitate the interpretation of the figure. PERMANOVA r-squared and *p*-values are shown. **d** Alpha diversity based on Shannon entropy index (ASV level) of individual fecal samples obtained at the last point of the study from small intestine, caecum and large intestine. Boxplots are colored by the experimental group. Median values and interquartile ranges are shown in boxplots. *p-*values from Wilcoxon signed rank tests are reported.

At family-level, the overall microbial community composition between Mock and Immunized groups was similar over the experiment (Fig. 2a). Samples from both fecal and luminal content were dominated by *Muribaculaceae* (49.8% and 57.9%)*, Lachnospiraceae* (14.2% and 11.4%)*, Bacteroidaceae* (9.4% and 4.8%) and *Bifidobacteriaceae* (7.1% and 6.2%) (Fig. 2a and Supplementary Fig. 6a), except for the small intestine (Supplementary Fig. 6b). In this gut section, reduction in *Lachnospiraceae NK4A136* group (0.2%) and *Bacteroides* (0.05%) was concomitant to an overall increase in *Lactobacillus* (7.7%) and *Ligilactobacillus* (3.7%) (Supplementary Fig. 6c, d). Despite overall compositional similarities during the vaccine administration (0 week–15 week, Fig. 2a and Supplementary Fig. 6e), samples clustered separately by experimental group (*p* = 0.022), but not by timepoint within each group (*p* = 0.594) (Fig. 2b), as indicated by both univariate and multivariate analyses (Supplementary Table 5). Cage and Sex covariates also showed a significant impact on the microbiota composition (Supplementary Table 5). Despite the lack of individual-level contribution in our assessments, a trend toward higher Bray–Curtis dissimilarity in the Immunized compared to the Mock group was observed (Supplementary Fig. 6f), potentially indicating a strong impact on the gut microbiota of vaccinated mice. Also, no differences in alpha diversity (Shannon index) were observed between groups during vaccination (Supplementary Fig. 6g). Clustering by experimental group (*p* = 0.021) and intestinal section (*p* = 0.001) was also observed at the last time point of the study, being samples from small intestine separated from those from caecum and large intestine (Fig. 2c). Moreover, samples from the small intestine showed lower alpha diversity in both Mock and Immunized groups when compared to caecum and large intestine, although no differences between the two experimental groups were detected (Fig. 2d).

### Gut microbiota alterations associated to HTI vaccination

**Fig. 3:**
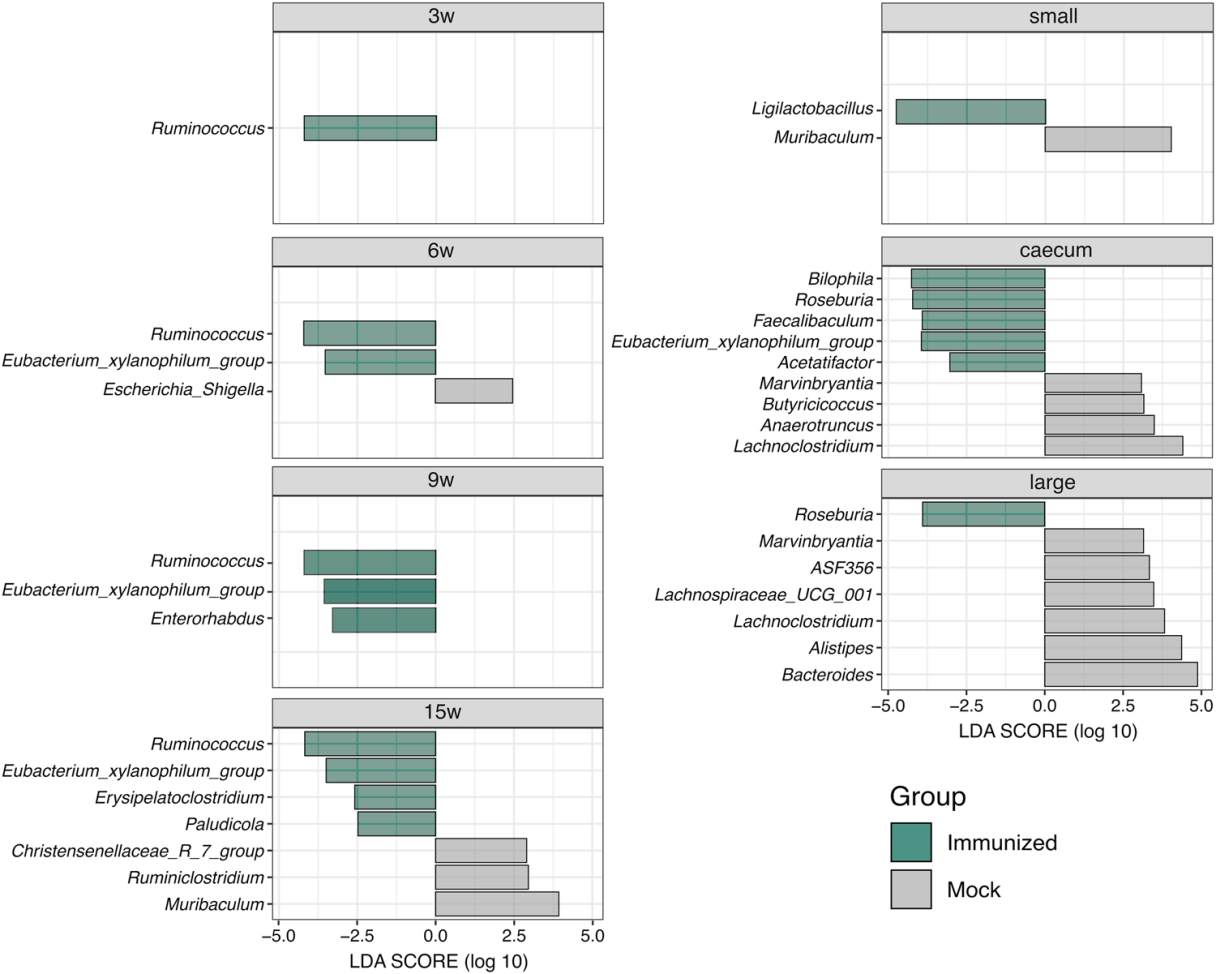
Discriminative genera between HTI- and PBS-immunized mice following vaccination. LEfSe analysis comparing the relative abundance of bacterial genera between Immunized and Mock groups during vaccine administration (pooled fecal samples at 3 week, 9 week, 6 week and 15 week) and at end of study at week 15 for individual fecal samples from small intestine, caecum and large intestine. Horizontal bars represent the effect size for each discriminant genus (*p* < 0.05 and LDA scores > 2.0). The bar length represents the log10 transformed LDA score, indicated by vertical lines.

The longitudinal discriminant analysis showed that the abundance of specific bacteria changed after vaccination. While only the *A2* genus was differentially abundant between experimental groups at the baseline (Supplementary Fig. 5a), the Immunized group showed an enrichment in a set of *Clostridiales* genera after vaccination (Fig. 3). The most remarkable observation was an enrichment in *Ruminococcus* in fecal samples from cages (week 3–15) and *Roseburia* in gut sections (caecum and large intestine), while *Eubacterium xylanophilum* group remained consistently higher in fecal samples from cages and gut sections after week 3. Also, *Ligilactobacillus* was increased specifically in samples from the small intestine (Fig. 3). Longitudinal assessment of such genera abundance indicated an increasing trend starting at vaccination in the Immunized group (Supplementary Fig. 7). Conversely, no specific genera were consistently higher in the Mock group over time.

### Correlations between gut microbial signatures and immunological responses

To examine the HTI immunogenicity, IFNγ assay assessing magnitude and breadth of the specific T cell response against HTI peptides was performed in splenocytes collected at the last time point of the study. No differences in the background IFNγ-producing splenocytes were found between Mock and Immunized mice, indicating that the vaccination regimen did not induce noticeable unspecific T cell activation (Supplementary Fig. 8a). After specific re-stimulation with peptide pools covering the HTI sequence, Mock group did not show any IFNγ response above the thresholds. In contrast, Immunized mice showed strong responses, in both magnitude (median = 1560 IFN-γ SFC/million splenocytes, *p* = 0.00031, Supplementary Fig. 8b) and breadth (median = 8.0 out of 17 positive pools, *p* = 0.00028, Supplementary Fig. 8c) compared to Mock.

**Fig. 4:**
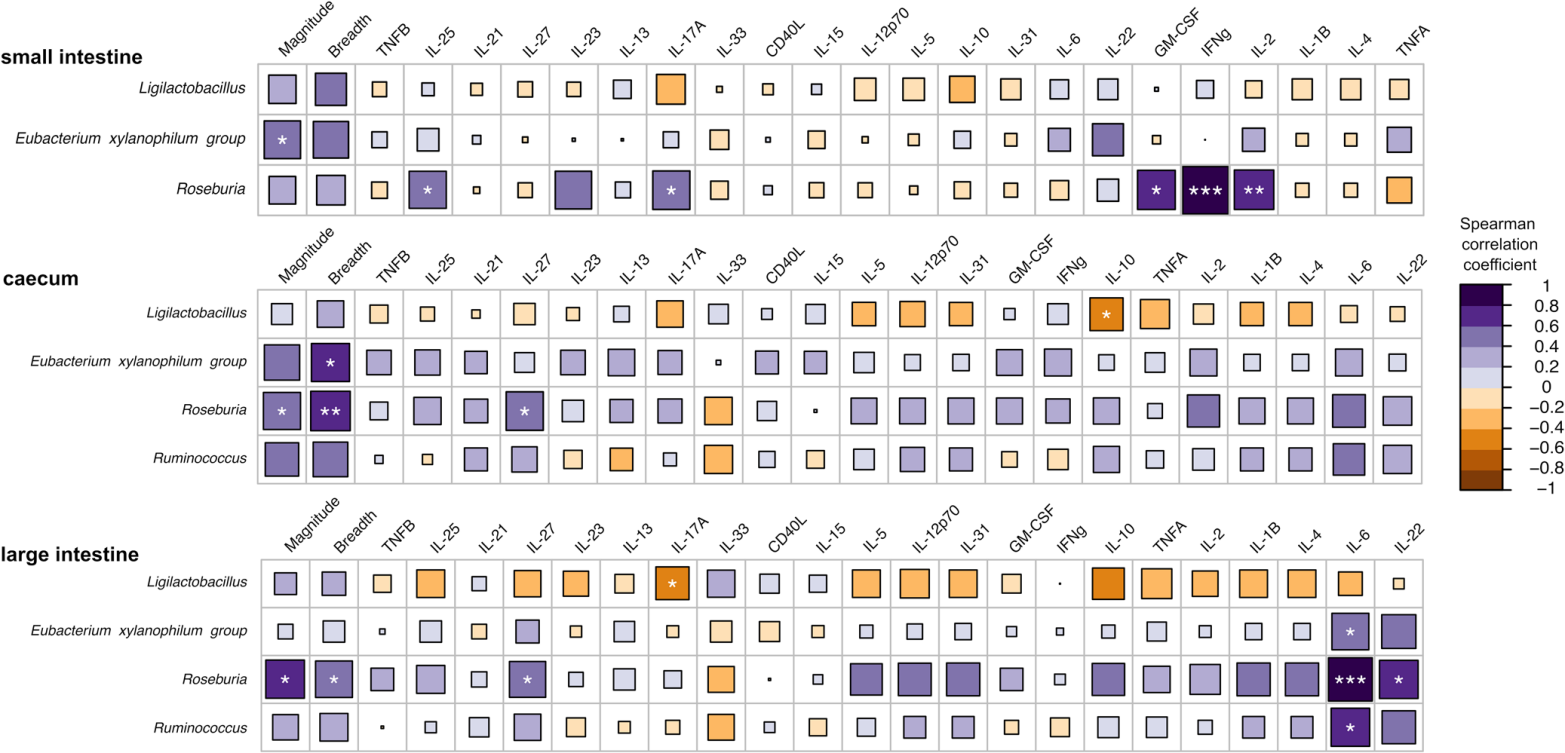
Correlation analysis between discriminant bacteria, serum cytokines and immunological responses at the last time point of the study. Spearman rank correlations between bacterial genera (only bacteria longitudinally enriched in HTI-immunized mice were evaluated), HTI immunogenicity (magnitude and breadth of the response) and serum cytokine levels. Associations are assessed for each gut section (small intestine, caecum and large intestine). Cytokines for each gut section are ordered based on unsupervised hierarchical clustering. Color and size of the square represent the magnitude of the correlation. Significance is indicated by white asterisks (*p < 0.05; **p < 0.01; ***p < 0.001 after Benjamini–Hochberg adjustment for multiple comparisons).

After assessing serum levels of 22 cytokines in Mock and Immunized groups (Supplementary Fig. 9a), IL-6 was the only cytokine showing an increasing trend in the Immunized group after segregating by sex (*p* = 0.052) (Supplementary Fig. 9b), although significance did not pass multiple testing correction (BH-adjusted *p* = 0.69). Specifically, females from the Immunized group showed lower IL-6 levels than males in both Immunized and Mock groups (Supplementary Fig. 9b). We next examined potential links between HTI-specific responses, serum cytokine levels and discriminant bacterial genera. *Eubacterium xylanophilum* group and *Roseburia* from the three intestinal sections were positively correlated with vaccine-induced HTI-specific T cell response (Fig. 4), measured as magnitude and breadth. In addition, most of the tested cytokines showed positive correlations with *Eubacterium xylanophilum* group*, Roseburia* and *Ruminococcus*, although there were some exceptions (i.e., IL-33). An opposite trend was observed for *Ligilactobacillus*, which overall showed negative associations with the serum cytokine levels (Fig. 4). Of note, IL-6 was positively associated with *Eubacterium xylanophilum* group*, Ruminococcus* and especially *Roseburia* in the large intestine. Moreover, *Roseburia* showed positive correlations with other cytokines, including GM-CSF, IL-2, IFNγ, IL-17A and IL-25 (small intestine), IL-27 (caecum and large intestine) and IL-22 (large intestine). Significant correlations were confirmed by individual scatterplots (Supplementary Fig. 10). Moreover, *Eubacterium xylanophilum* group*, Roseburia* and *Ruminococcus* from the three intestinal compartments clustered close to the HTI-specific responses (magnitude and breadth) in a hierarchical clustering, also displaying positive associations with a group of cytokines (Supplementary Fig. 11).

## Discussion

To date, limited studies have investigated how vaccine administration may alter resident gut microbial communities. Herein, we used a prime-boost regimen combining the HTI insert in different vaccine vectors, to induce strong HTI-specific responses and study their impact on the microbiota. These results showed, in mice, an increase in *Clostridiales* bacteria following HIV T-cell immunization, which in turn associate with proinflammatory cytokines. Except for *Eubacterium xylanophilum* group, *Clostridiales* genera found in feces were different from those in the three gut sections in the Immunized group. Such differences might be related to the presence of distinct species in the intestinal epithelium compared to the lumen^24^ and the presence of a 50-60% spore forming bacteria, like *Ruminococcaceae* and *Lachnospiraceae*, most likely to be present in feces^25^. Remarkably, associations between enriched bacteria and serum cytokines varied across the three intestinal sections, being representative of distinct oxygen requirements and a different crosstalk between mucosal cells and co-existing microbial communities^26^. The most prominent difference was observed in the small intestine, which was depleted in obligate anaerobes bacteria, such as *Ruminococcacae* and *Lachnospiraceae*, compared to the caecum and large intestine, as previously reported^27^.

Supporting our results, a recent study in rhesus macaques showed that vaccination with three HIV-1 Env expressing DNA plasmid vaccines followed by a gp140 protein booster induced changes in the rectal microbiota, resulting in increased *Firmicutes/Bacteroidetes* ratio as well as associations of rectal anti-HIV-1 IgG vaccine responses with increased *Clostridium IV* and reduced *Prevotella*^17^. Enrichment in *Ruminococcus* in the intestinal microbiota of piglets was also reported after immunization with *Lawsonia intracellularis* vaccine^28^. In parallel, increased *Bacteroides* abundance along with alterations in the microbial community structure were found following Mtb vaccine (with ESAT6 adjuvanted with TLR8 agonists) administration in mice^29^. Similarly, an enrichment in *Bacteroides caccae* and reduction in clostridial species, such as *Coprococcus comes, Dorea longicatena and Ruminococcus obeum* were reported in Covid-19 vaccinees^18^. Such apparently conflictive trends indicate that a more thorough understanding is needed to decipher specific microbial shifts induced by distinct immunization strategies.

Our data indicated that bacteria increased after HTI vaccination (*Eubacterium xylanophilum* group*, Roseburia* and *Ruminococcus*), located in the three distinct gut sections, positively correlated with HTI-specific T cell response and a group of serum cytokines. Specifically, the genus *Roseburia*, known as a butyrate-producing bacteria^30^, in the caecum and large intestine, positively correlated with the magnitude and breadth of HTI specific T-cell response in spleen cells. At serum level, *Roseburia* correlated with IL-27, which is produced by antigen presenting cells and regulates B and T cells^31^. Interestingly, despite its different effector function, IL-27 shares the gp130 receptor subunit with IL-6^32^ and both cytokines and IL-22 were associated with the content of *Roseburia* in the large intestine. Although the levels of *Roseburia* in small intestine did not correlate with HTI-specific T cell responses, strong correlations were observed with inflammatory cytokines (IL-6, GM-CSF), two Th1 polarization cytokines (IFNγ and IL-2) and the Th17 polarization cytokines (IL-17A and IL-22). However, none of these cytokines were differentially produced between the Mock and the Immunized group and only IL-6 showed a marginal significance, which did not pass multiple comparison correction. We hypothesize that the abundance of *Roseburia* may increase due to T cell vaccine stimulation and relate to Th1 responses, as indicated by positive correlation with IFNγ and IL-2 in plasma, but most remarkably with the HTI-specific T cell responses in spleen cells. The increase in *Roseburia* may be also related to the presence of inflammation mediators such as IL-6^33^, likely produced after vaccination and Th17 (IL-17A and IL-22) cytokines in serum, which could be affecting mucosal immune responses in the gut. Additional butyrate producing *Clostridiales* genera^34^ enriched in vaccinated animals showed positive correlations with the HTI-specific T cell responses (*Eubacterium xylanophilum* group in small intestine and caecum) and serum levels of IL-6 (*Ruminococcus* and *Eubacterium xylanophilum* group in large intestine). Also, although *Ligilactibacillus* did not associate with T cell responses, correlations with cytokines representatives of Th17 and Treg population (IL-10 and IL17A), known as gut inflammation biomarkers^35^, might indicate specific changes in the microbiota composition. While the exact mechanism by which HTI-immunization would induce changes in the intestinal milieu and promote an increase of specific bacteria remains unclear, cytokine signaling after innate immunity and T cell activation by vaccination is a plausible hypothesis. In fact, intramuscular vaccine administration may induce local ‘depot’ effect. This consists in a prolonged antigen release at the site of injection, which induce persistent stimulation of the immune system and recruitment of macrophages that, in turn trigger activation of inflammatory mediators^36^. Of note, administration of viral vaccine vectors rich in Pathogen Associated Molecular Patterns (PAMP), as used in this study, can also trigger the activation of Pathogen Recognition Receptors (PRRs), stimulating the production of pro-inflammatory cytokines, such as IL-1 and TNF-α by dendritic cells and macrophages^37^. Inflammation mediators can then migrate through the bloodstream and lymph vessels to reach intestinal tissues and promote local changes in the immune response and, in turn, alter the associated microbiota structure^38^. Furthermore, immune cells, such as dendritic cells or regulatory T cells, activated at the site of infection, can mediate inflammatory signaling to the lymph nodes, including mesenteric lymph nodes or the Peyer Patches^39^.

Notably, bacteria correlated with pro-inflammatory cytokines (such as IL-6, IL-27 and IFNγ) in our study (*Roseburia*, *Ruminococcus* and *Eubacterium*) are known as primary contributors of anti-inflammatory metabolites, mainly short chain fatty acids (SCFAs), in the gut^40^. This finding may appear inconsistent with previous evidence, such as in inflammatory bowel diseases, in which SCFAs inhibit the production of pro-inflammatory cytokines^41^; although, several mechanisms by which commensal bacteria can adapt to inflamed gut environment have been discussed elsewhere^42^. For example, specific components of the gut microbiota have been implicated in the production of pleiotropic cytokines by innate immune cells, such as IL-6, and subsequent expansion of Th17 cells^43^, which are critical for vaccine-induced memory immune responses^44^. Also, flagellin and peptidoglycans produced by gut bacteria, such as *Ruminococcus* spp.^45^, can be sensed by pattern recognition receptors (PRRs) expressed by T cells and B cells and act as natural adjuvants to vaccines^7^. In fact, higher abundances of bacteria with flagella and fimbriae, including butyrate-producing such as *Roseburia* and *Eubacterium*, can boost vaccine immunogenicity serving as adjuvants through immunomodulatory TLR agonists^46^.

Notably, members of these bacterial groups, such as *Roseburia intestinalis* and *Eubacterium hallii* have been listed as probiotic candidates by the International Scientific Association for Probiotics and Prebiotics^47^. Indeed, some butyrate-producing bacteria, including those identified in this study, have been shown to modulate the colonic luminal metabolome, regulate T-cell responses and enhance regulatory T-cell functions^48^. Collectively, these data prompted the hypothesis that bacteria producing anti-inflammatory molecules may adapt to increased local inflammation following vaccination, colonize the intestinal milieu and thrive in it, thereby modulating and quenching the inflammatory processes. However, further work is needed to understand how vaccine-induced inflammation promotes an increase of anti-inflammatory bacteria in the gut. In this context, Th17/Treg cell balance, T cell polarization and activation profiles, ILC responses and Kyn/Trp ratio^49^ in the gut tissues are promising candidates for future validations. Also, additional omics approaches, including metabolite and targeted SCFA measurements, would help to identify potential anti-inflammatory pathways induced after HTI vaccination. We fully acknowledge the limitation of the small sample size and lack of individual-level contribution in this study. Also, the lack of longitudinal cytokine measurement did not allow us to identify temporal variations of immune correlates with specific bacterial signatures at each stage of the vaccine regimen. Despite a group of bacteria showed strong correlations with HTI-specific T cell responses, another limitation was that the experimental design did not allow to differentiate to which extent vaccine-induced microbiota changes were due to an adjuvant effect of the vectors or to insert-specific responses. Future experiments including a control arm using empty vectors will help to discriminate the direct contribution of the vaccine insert on gut microbiota changes. In addition, another intrinsic limitation was the inability of the 16 S rRNA gene sequencing to distinguish live organisms from transient microorganism colonization^50^. Finally, host-specificity of the core gut microbiota may limit potential extrapolation of these results from a mouse model to humans. Thus, clinical trials including microbiota characterization are needed to validate such findings in the human gut microbiota.

In summary, our data suggest that gut bacteria can adapt to vaccine-induced inflammation. Also, they establish a framework for future studies to fully capture the dynamics of microbial shifts to T-cell vaccination and, ultimately provide new potential targets for optimizing vaccine efficacy.

## Methods

### Mice and housing

Six-week-old C57BL/6JOlaHsd mice (*n* = 40, 20 females and 20 males), grown in the same breeding facility, were purchased from Envigo and housed under specific pathogen-free (SPF) conditions at the Centre for Comparative Medicine and Bioimage (CMCiB) animal facility at IGTP in Badalona, Spain. Mice were randomly assigned to cages according to a combination of sex (female/male) and treatment group (Mock/Immunized), with a maximum of 5 animals per cage (Supplementary Fig. 1) and allowed to acclimate for one week before the initial treatment.

### Pre-intervention gut microbial homogenization

To reduce potential baseline differences in the microbiota composition, the mice gut microbiota was homogenized by antibiotic conditioning followed by mouse-to-mouse FMT (1 week after arrival and 2 weeks prior to vaccination). The antibiotic treatment consisted of a combination of ampicillin, amikacin, metronidazole and vancomycin (10 mg/ml each antibiotic), administered via oral gavage (200 µl/animal) for 5 days prior to FMT. Mixed stools from five male and five female donor mice (cages MF1 and MM1, Supplementary Fig. 1) were used for FMT in all animals in the study. Briefly, feces from donors were collected, pooled, aliquoted (350 mg vials) and preserved at −80 °C until use. On the day of the fecal transfer, thawed feces were resuspended in sterile physiological saline serum (2.3 ml per vial), homogenized, centrifuged (500 × g, 1 min) and supernatant administered by oral gavage (100 µl/animal). Bacterial load in FMT was measured using the LIVE/DEAD^®^ BacLight™ Bacterial Viability and Counting Kit (Invitrogen, Carlsbad, MA, USA).

### Immunization strategy

Animals were vaccinated with the HIV T-cell immunogen (HTI), a synthetic protein designed as the fusion of different HIV-1 protein fragments associated with T-cell control of HIV infection^20^. Briefly, the HTI sequence encodes for a 529 amino acid immunogen, designed as the concatenation of 16 clade B consensus HIV Gag, Pol, Vif and Nef protein fragments, which were preferentially targeted by people showing natural control of HIV infection, linked by alanine triplets. The amino acid sequence was back-translated into nucleotides, codon usage optimized for expression in human and the resulting open reading frame (ORF) synthetized to be inserted in different vectors^22^. Specifically, it was inserted into a plasmid DNA under the control of a CMV promotor (DNA.HTI, D) and into two viral vectors: Chimpanzee Adenovirus Ox1 (ChAdOx1.HTI, C) and Modified Vaccinia Ankara (MVA.HTI, M)^22^. HTI vaccine immunogenicity has been previously tested in C57BL/6 mouse model and non-human primates^22^ as well as in humans^23^. In this study, twenty mice (10 females and 10 males) were immunized intramuscularly (caudal thigh muscle) with a heterologous prime-boost regime consisting of three different vaccine vectors expressing HTI: (i) three DNA.HTI primes (100 μg/animal), (ii) one boost with ChAd.HTI (1 × 10^9^ VP/animal), and (iii) a second boost with MVA.HTI (1 × 10^6^ pfu/animal). DNA.HTI vaccinations were administered at weeks 0, 3, and 6, followed by ChAd.HTI and MVA.HTI, separated by a 6-week interval (weeks 9 and 15, respectively) (Fig. 1 and Supplementary Fig. 1). Mice in the mock group (*n* = 20, 10 females and 10 males) were vaccinated with PBS as a control group, following the same immunization schedule.

### Sample collection and processing

Microbiota data were generated in two batches (samples extracted and sequenced in 2019 and 2020), as illustrated in Supplementary Figs. 1 and 2. The first sequencing batch included pooled fecal samples longitudinally collected from cages, whereas the second batch included longitudinal pools of feces from cages and individual fecal samples obtained from different sections of the intestinal tract at the last time point of the study. Longitudinal fecal samples were collected (one pool per cage, *n* = 5) at animal arrival (week-3), following antibiotic conditioning prior to FMT (week-2) and prior each vaccination (weeks 0, 3, 6, 9 and 15). Donor pool fecal sample (week-1) was also collected and sequenced in batch 1. Three weeks after the last immunization (week 18), mice were euthanized and luminal contents from three intestinal sections (small, caecum and large intestine), whole blood and spleen were harvested. Serum was separated from the whole blood by centrifugation (10,000 × *g*, 5 min) in Serum Gel S/1.1 tubes (Sarstedt) and frozen at −80 °C until use. Splenocytes were isolated by mechanical disruption and pressed through a cell strainer (Falcon) using a 5 ml-syringe rubber plunger. Following red blood cell lysis, splenocytes were washed, resuspended in R10 (RPMI 1640 supplemented with 10% fetal bovine serum, 2 mM L-glutamine, 100 U/ml penicillin and 100 µg/ml streptomycin) and cryopreserved in liquid N_2_ until use.

### DNA extraction and 16 S rRNA sequencing

Genomic DNA from fecal samples and intestinal content was extracted using the QIAamp DNA Stool Kit, according to the manufacturer’s instructions, and stored at −80 °C until sequencing. Amplification of the V3–V4 hypervariable region of the 16 S rRNA gene was performed using universal primers flanked by standard forward and reverse adapters (16S_F 5′-TCG TCG TCG GCA GCG TCA GAT GTG TAT AAG AGA CAG CCT ACG GGN GGC WGC AG-3′; 16S_R 5′-GTC TCG TGG GCT CGG AGA TGT GTA TAA GAG ACA GGA CTA CHV GGG TAT CTA ATC C-3′) described in the Illumina MiSeq rRNA Amplicon Sequencing protocol (San Diego, CA, USA). PCR were performed in 25 μL reaction volume, containing 2.5 μL of DNA template, 12.5 μL of KAPA HiFi HotStart Ready Mix (KAPA HiFi HotStart DNA Polymerase, buffer, MgCl_2_, and dNTPs, KAPA Biosystems Inc., Wilmington, MA, USA) and 5 μL of each primer at 1 μM. Thermocycling conditions were as follows: initial denaturation step at 95 °C for 3 min, followed by 30 cycles of denaturation at 95 °C for 30 s, annealing at 55 °C for 30 s, extension at 72 °C for 30 s and a final extension step at 72 °C for 10 min. Once the expected amplicon size was confirmed on a 1.0% agarose gel electrophoresis (~460 base pairs), PCR products were stored at −30 °C until sequencing library preparation. Negative extraction and PCR controls (blank controls using DNA-free water as the template) were loaded to assess for potential contamination and processed in the same conditions as any other sample. Amplified DNA templates were cleaned-up with AMPure XP reagent (Beckman Coulter Life Sciences, Indianapolis, USA) for non-DNA molecules and Illumina sequencing adapters and dual indices were attached using Nextera XT index Kit (Illumina Inc.), followed by a corresponding PCR amplification program as described in MiSeq 16 S rRNA Amplicon Sequencing protocol (Illumina Inc., San Diego, CA, USA). After the second round of clean-up, amplicon libraries were quantified using a Quant-iT^TM^ PicoGreen^®^ dsDNA Assay Kit (Invitrogen, Carlsbad, MA, USA) and diluted in equimolar concentrations (4 nM) for further pooling. Pooled libraries were sequenced on an Illumina MiSeq^TM^ platform (Illumina Inc., San Diego, CA, USA) at the genomics core facility in Germans Trias i Pujol research campus (Badalona, Spain) using a paired-end 300 base-length protocol.

### Microbiome data analysis

The quality of raw sequencing data was estimated using FastQC^51^ and the analysis of 16 S rRNA sequences performed using the DADA2 pipeline (v1.10.1)^52^. The pipeline was executed according to default parameters using maxEE = 4.10 in the filtering step. After chimeric reads removal, unique amplicon sequence variants (ASV) were assigned a taxonomy and aligned to the SILVA rRNA gene database^53^. Downstream analyses were conducted in the R environment (v3.5.2)^54^ using multiple packages, including *phyloseq* (v1.26.1)^55^, *vegan* (v2.5-5)^56^ *ade4* (v1.7-13)^57^ and *ggplot*2 (v3.2.0)^58^. ASV counts were normalized and transformed to relative abundance using the phyloseq function *transform_sample_counts* (100 × (x/sum(x))). Alpha diversity (Shannon entropy index) was evaluated using the *estimate_richness* on rarefied ASV counts. Beta diversity was calculated based on Bray–Curtis distances (normalized data) and distance matrix used to perform an ordination principal coordinate analysis (PCoA).

### Mouse IFNγ ELISPOT assay

ELISPOT assays were performed using the mouse IFNγ ELISPOT kit (ALP) (Mabtech AB, Stockholm, SE), following the manufacturer’s instructions with minor modifications. Briefly, splenocytes were thawed, washed, counted (NucleoCounter® NC-3000, Chemometec), cell density adjusted with R10 at 4 × 10^5^ cells/well and plated in 96-well polyvinylidene plates (Millipore Corp., Bedford, MA), previously coated with IFNγ capture antibody (clone AN-18). Cells were stimulated with 17 HTI- specific peptide pools (14 μg/ml final concentration for each peptide) for 16 h at 37 °C in 5% CO2. A set of 147 overlapping peptides (OLP) of 15 amino acids in length, overlapping by 11 amino acids and covering the entire HTI sequence, were designed using the PeptGen algorithm (Los Alamos HIV database) and synthesized (Synepeptide). To assess responses to HTI, OLP were assembled into 17 separated peptide pools consisting of 7 pools for Gag (8-11 peptides/pool), 7 for Pol (11 or 5 peptides/ pool), 2 for Vif (8 and 6 peptides/pool) and 1 for Nef (2 peptides/pool). Concanavalin A (Sigma-Aldrich Corp., Saint Louis, MO), at 5 µg/ml, was used as a positive control and R10 in triplicate as a negative control. After stimulation, spot-forming cells (SFC) were revealed by adding a biotinylated IFNγ detection antibody (clone R4-6A2), streptavidin conjugated with alkaline phosphatase (AP) and AP Conjugate Substrate Kit (Bio-Rad Laboratories, Inc., Irvine, CA). SFC per well were counted using an automated ELISPOT reader system (CTL Analyzers LLC, Cleveland, OH) together with the ImmunoSpot software and the magnitude of responses expressed as SFC per 10^6^ splenocytes. Threshold for positive responses was defined as responses that were at least 50 SFC/10^6^ spleen cells, the mean SFC/10^6^ spleen cells in negative control wells plus 3 standard deviations of the negative control wells, or three times the mean of negative control wells, whichever was higher. Results were given as total magnitude (accumulated response to all the pools; SFC/10^6^ PBMCs) and breadth (number of positive pools).

### Luminex assay

Concentrations of CD40 ligand (CD154), GM-CSF, IFN γ, IL-1ß, IL-2, IL-4, IL-5, IL-6, IL-10, IL-12 (p70), IL-13, IL-15, IL-17A, IL-17E/IL-25, IL-21, IL-22, IL-23, IL-27, IL-31, IL-33, TNFα, TNFß at the end of the study (week 21 wpi) were simultaneously measured in mice sera, in a single batch. Briefly, serum samples were thawed, vortexed and centrifuged at 10000 xg for 10 min before collecting 25 µl of serum for further testing. The serum concentration of the 22 cytokines was measured simultaneously using sample duplicates in a customized mouse Th17 magnetic bead panel kit (Milliplex) and a Luminex^®^ 200 (Luminex Corp) reader, following manufacturer’s instructions.

### Statistical analysis

Differences in alpha diversity and taxa abundance between experimental groups were assessed using the Wilcoxon signed-rank and Kruskal-Wallis test (two sided). For beta diversity, statistical significance was assessed using the pairwise permutational multivariate analysis of variance (PERMANOVA, *adonis*) test. The linear discriminant analysis effect size (LEfSe)^59^ was performed to identify discriminant bacterial signatures between Mock and Immunized groups (*α* = 0.05 and LDA score > 2.0). Associations between cytokines, immune response and bacteria were computed based on Spearman correlation coefficients in combination with Benjamini–Hochberg multiple testing correction using the *rcorr* function within the R/hmisc package. Statistical analyses were performed in the R environment and two-tailed 5% level of significance (*p*-value **≤** 0.05) was considered significant, unless otherwise stated.

### Reporting summary

Further information on research design is available in the Nature Research Reporting Summary linked to this article.

## Supplementary information

Supplementary Material

Reporting Summary

Supplemental Tables

## Data Availability

The raw 16 S rRNA sequencing reads used in this study have been deposited at the Sequence Read Archive (SRA) repository (Bioproject No. PRJEB52963).

## Code availability

R analysis scripts and data associated with this paper are available at 10.5281/zenodo.7017193.
