# Supplementary material for: Vaccination with an HIV T-cell immunogen induces alterations in the mouse gut microbiota
Source: NPJ Biofilms Microbiomes. 2022 Dec 30;8:104. doi: 10.1038/s41522-022-00368-y (PMC9801356; doi:10.1038/s41522-022-00368-y)
Supplement: Supplementary file 1 — Supplementary Material [file 41522_2022_368_MOESM1_ESM.pdf]

Supplementary File for:

Vaccination with an HIV T-cell immunogen induces alterations in the mouse gut microbiota

Alessandra Borgognone[1], Aleix Elizalde-Torrent[1], Maria Casadellà[1], Luis Romero[1,2], Tuixent Escribà[1], Mariona Parera[1], Francesc Català-Moll[1], Marc Noguera-Julian[1,3,4], Christian Brander[1,3,4,5,6], Alex Olvera[1,3,4,*], Roger Paredes[1,2,3,4,7,8,9,*]

[1] IrsiCaixa - AIDS Research Institute, Badalona, Spain.

[2] Universitat Autònoma de Barcelona (UAB), Barcelona, Catalonia,Spain

[3] Universitat de Vic-Universitat Central de Catalunya (UVic-UCC), Vic, Spain.

[4] CIBERINFEC - ISCIII

[5] Institució Catalana de Recerca i Estudis Avançats (ICREA), Barcelona, Spain.

[6] AELIX Therapeutics, Barcelona, Spain

[7] Center for Global Health and Diseases, Department of Pathology, Case Western Reserve University, Cleveland, OH, United States of America

[8] Fundació lluita contra les Infeccions, Hospital Universitari Germans Trias i Pujol, Badalona, Catalonia, Spain

[9] Department of Infectious Diseases, Hospital Universitari Germans Trias i Pujol, Badalona, Catalonia, Spain

This Word file includes:

Supplementary Text

Supplementary Figures

Supplementary Text

Effects of antibiotic conditioning and FMT on gut microbiota populations

Antibiotic pre-treatment followed by mouse-to-mouse FMT was performed to homogenize the intestinal content across experimental groups before vaccine administration[1]. As expected in animals with the same origin, the mice gut microbiota at arrival (pool of feces obtained from different cages) showed high similarity (-3w, Supplementary Fig. 2a) with a clear dominance of *Muribaculaceae* (44.3 % global average), followed by *Lachnospiraceae* (14.01%), *Bacteroidaceae* (13.04%), *Bifidobacteriaceae* (8.1%) and *Lactobacillaceae* (6.2%). A shift in bacteria composition (-2w, Supplementary Fig. 2a) and significant decrease in alpha diversity (p-value = 0.029, Supplementary Fig. 2b) were observed after antibiotic conditioning. In particular, the abundance of *Muribaculaceae* was drastically reduced (5.5% global average), with a concomitant increase in *Bacteroidaceae* (29.6%), *Enterobacteriales* (24.6%), *Tannerellaceae* (15.05%). Also, *Akkermansia* was detected in male mice on average at 9.6% abundance (-2w, Supplementary Fig. 2a). Due to its clinical relevance, especially in obesity, inflammation and cancer treatment[2], specific searches for the genus *Akkermansia* were conducted. Overall, this genus was present in low abundance (global mean = 0.45%, range = 0-22%), except for some male mice after antibiotic treatment and FMT. Of note, changes in *Akkermansia* abundance after antibiotic treatment have already been reported[3]. In this study, we did not detect significant differences in *Akkermansia* between groups, suggesting that this genus was not altered after vaccination. Although, it remains undefined if this point could be extrapolated to humans, in which *Akkermansia muciniphila* might play a different role in the gut microbial community. Fecal sample used as microbiota donor (-1w, FMT-Donor) showed high similarity in composition to samples at arrival (46.7 % *Muribaculaceae*, 13.4% *Lachnospiraceae*, 11.8% *Bifidobacteriaceae*, 7.7% *Bacteroidaceae* and 6.8% *Lactobacillaceae*, Supplementary Fig. 2a). Microbiota homogenization among the animals included in the study was observed one week after FMT, suggesting FMT engraftment, with microbial communities recovered across all samples (0w, Supplementary Fig. 2a). Such results were recapitulated by compositional dissimilarity analysis

(PCoA based on Bray-Curtis distances) showing that samples at arrival and post-FMT clustered together with the FMT-Donor, whereas samples after antibiotic treatment grouped separately ($p=0.001$ PERMANOVA, Supplementary Fig. 2c). Also, samples at arrival (-3w) and post-FMT (0w) did not show significant differences in composition (Supplementary Fig. 2a) and microbial diversity (Supplementary Figs. 2b and d), indicating that decrease in diversity caused by antibiotic pre-treatment was restored by FMT (Supplementary Fig. 2b). However, while the gut microbiome structure disrupted by antibiotic conditioning was successfully restored after FMT, samples post-FMT did not show higher homogenization (lower beta diversity) when compared to samples at arrival. Indeed, considering that the viability of the FMT was low (3.8%) and pre-FMT antibiotic conditioning did not completely depleted the microbiota, there is a chance that the gut microbiota was partially recovered from the pre-existing bacterial communities. Moreover, despite mouse-to-mouse FMT was performed to reduce sex-related variability by mixing female and male feces, differences in the gut microbiota prior vaccination were found (Supplementary Fig. 4b).

Of note, similar sex differences have been reported in previous studies, with female mice showing higher abundances of *Lactobacillus*, but not *Muribaculum* (in this study, *Muribaculum* abundance was low, < 1%)[4]. While it is difficult to identify specific factors influencing sex dimorphism in the gut microbiota, the biochemistry of circulating sex hormones, either directly or indirectly affecting gut microbes might be one of the mechanisms underlying these differences[4].

Considering these results, together with contamination issues intrinsically linked to stool collection and transferring[5] factors, such as sex dimorphism in the gut microbiota as well as animal welfare, interventions including FMT with antibiotics pre-conditioned microbiota might be not required in future studies implicating similar experimental designs.

References

1. Shang, L., Tu, J., Dai, Z., Zeng, X. & Qiao, S. Microbiota Transplantation in an Antibiotic-Induced Bacterial Depletion Mouse Model: Reproducible Establishment, Analysis, and Application. *Microorganisms* **10**, (2022).
2. Cani, P. D., Depommier, C., Derrien, M., Everard, A. & de Vos, W. M. Akkermansia muciniphila: paradigm for next-generation beneficial microorganisms. *Nature Reviews Gastroenterology & Hepatology 2022 19:10* **19**, 625–637 (2022).
3. Zhang, N. *et al.* Integrated Analysis of the Alterations in Gut Microbiota and Metabolites of Mice Induced After Long-Term Intervention With Different Antibiotics. *Front Microbiol* **13**, 2283 (2022).
4. McGee, J. S. & Huttenhower, C. Of mice and men and women: Sexual dimorphism of the gut microbiome. *International Journal of Women's Dermatology* vol. 7 (2021).
5. Bokoliya, S. C., Dorsett, Y., Panier, H. & Zhou, Y. Procedures for Fecal Microbiota Transplantation in Murine Microbiome Studies. *Frontiers in Cellular and Infection Microbiology* vol. 11 (2021).

Supplementary Figures

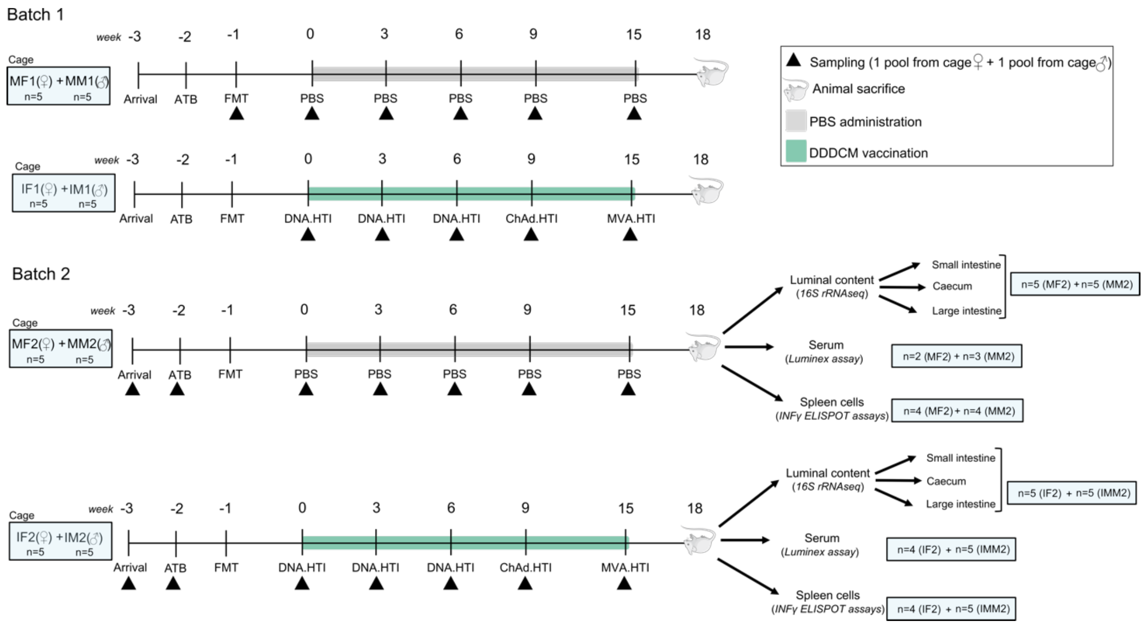

Supplementary Figure 1. Summary of the study design, sample collection and data production. Female and male mice were separated in cages (n=5 animals per cage) according to the experimental group (mock, M and immunized, I) and sex (female, F and male, M). HTI T cell vaccine (three DNA.HTI primes followed by a ChAd.HTI and MVA.HTI booster) or a mock vaccine (PBS) were administered as illustrated in the diagram. Prior vaccination (0w, baseline), mice guts were conditioned with antibiotics (-2w) followed by fecal microbiota transplant (-1w). Fecal donor sample was obtained by mixing feces from cages MF1 and MM1 at the animal arrival. Pooled fecal samples from cages were collected at the indicated timepoints (black triangles) and sequenced using a 16S rRNA sequencing approach in two batches (2019 and 2020). Intestinal content (small intestine, caecum and large intestine), serum and spleen samples were collected after sacrifice from each animal in batch 2 and processed for microbiota analysis and immunological response assessment. Abbreviations: IF=immunized females, IM=immunized males, MF=mock females, MM=mock males, FMT=fecal microbiota transplantation, ATB=Antibiotic conditioning, PBS=phosphate-buffered saline.

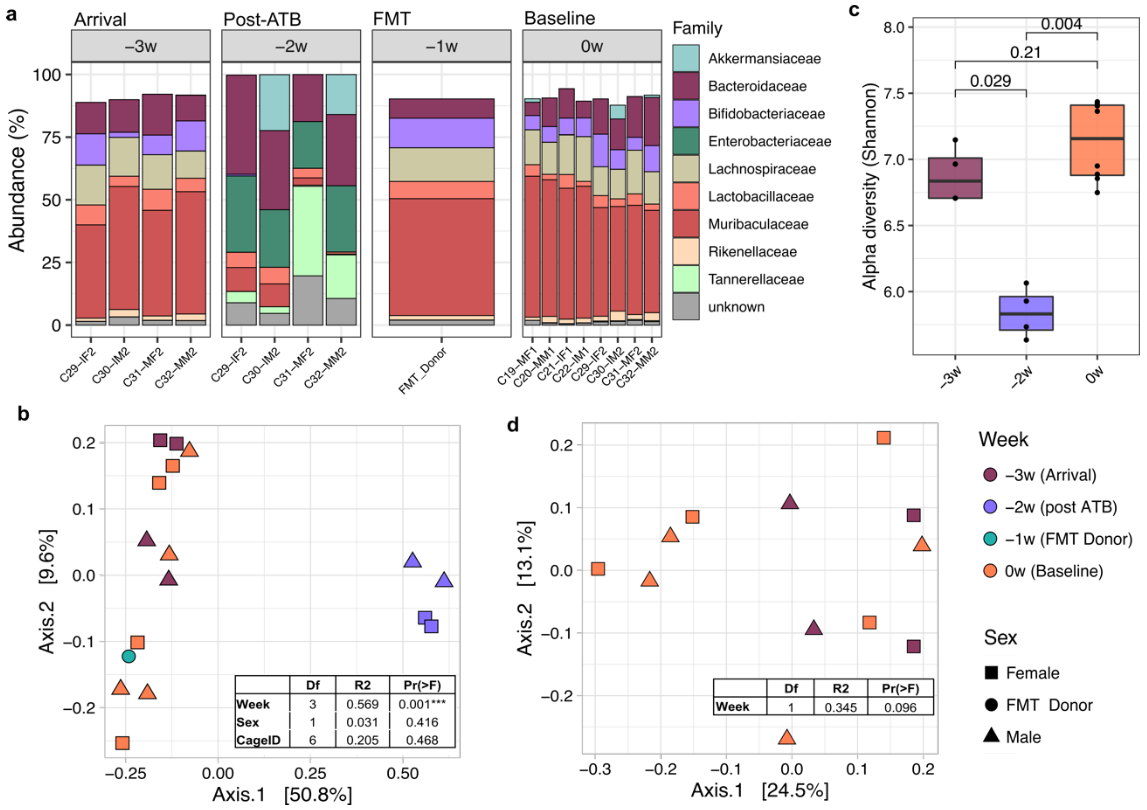

Supplementary Figure 2. Fecal microbiota profiling before and after FMT. (**a**) Stacked bar chart illustrating bacterial composition at the family level. Top 10 most abundant families are displayed. CageIDs and FMT-donor sample are reported in the *x*-axis and relative abundance (as percentage) of taxa in the *y*-axis. (**c**) Boxplots displaying alpha diversity (ASV level) of samples at arrival (-3w), post antibiotic conditioning (-2w) and one week after fecal microbiota transfer (0w, baseline). Median values and interquartile ranges are shown in boxplots. *p*-values from Wilcoxon signed rank test are reported. (**b**) PCoA of microbiota composition based on Bray-Curtis distances (ASV-level) from sample arrival to post-FMT (baseline) and (**d**) only at arrival and post-FMT. Each point represents a cage (pooled feces) or FMT-donor sample. The proportion of variance corresponding to each principal component is reported in the corresponding axis. PERMANOVA r-squared and p-values are shown. *Abbreviations*: FMT=fecal microbiota transfer, Post-ATB=After antibiotic conditioning.

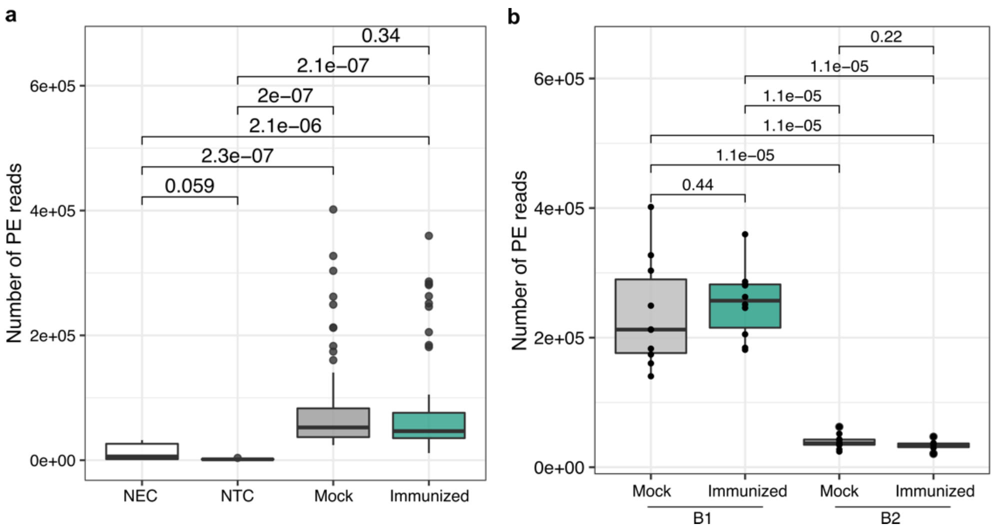

Supplementary Figure 3. Comparison of 16S rRNA sequencing yield. Boxplots showing filtered paired-end reads in (**a**) negative controls (NEC and NTC) and fecal samples (from cages and three intestinal sections) from Mock and Immunized mice. (**b**) Read counts comparison of pooled fecal samples from Mock and Immunized groups (w0-w15) processed in batch 1 (2019) and batch 2 (2020). Median values and interquartile ranges are shown in boxplots. *p*-values from Wilcoxon signed rank test are reported. *Abbreviations*: NEC=negative extraction controls, NTC= negative template controls, B1=sequencing batch1, B2=sequencing batch2.

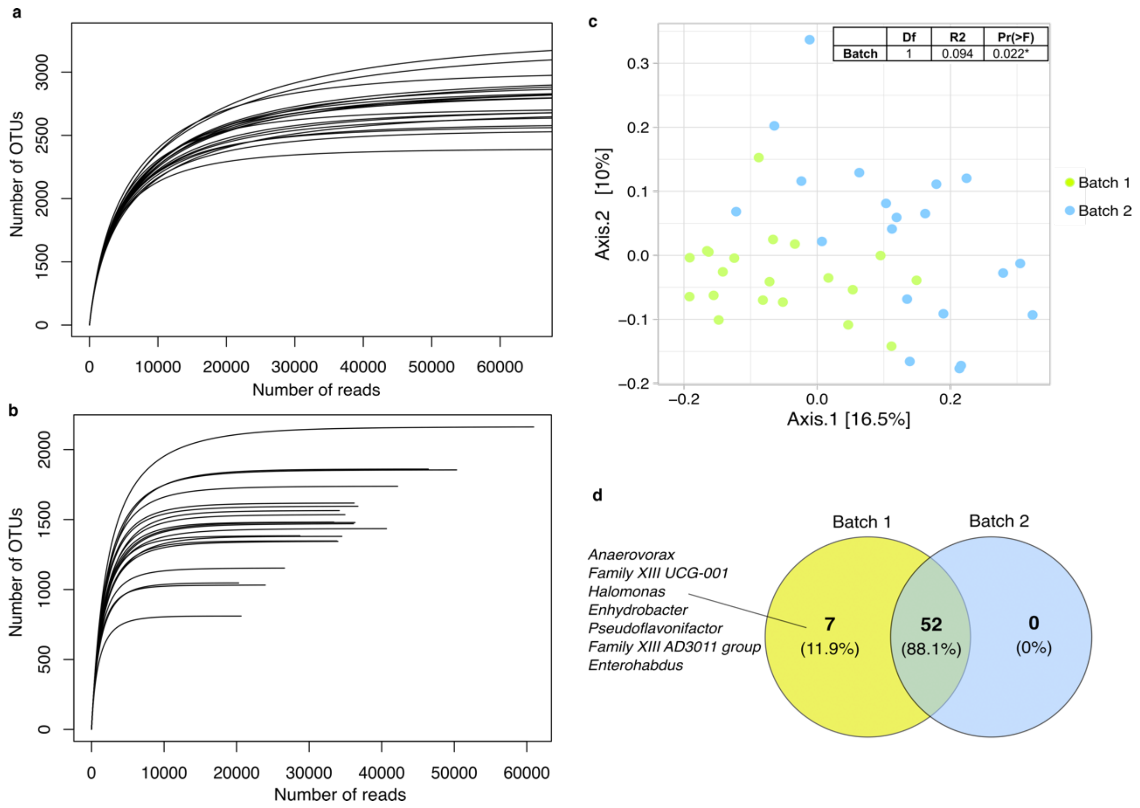

Supplementary Figure 4. Evaluation of sequencing batch covariate on data structure. Rarefaction curves for pooled fecal samples from cages in Batch 1 (a) and Batch 2 (**b**) at 60,000 number of reads. (**c**) PCoA based on Bray-Curtis distances for pooled fecal samples randomly rarefied at 20,000 number or reads. (**d**) Venn diagram of exclusive and shared bacterial genera between samples from Batch 1 and Batch 2 (random rarefaction at 20,000 number or reads).

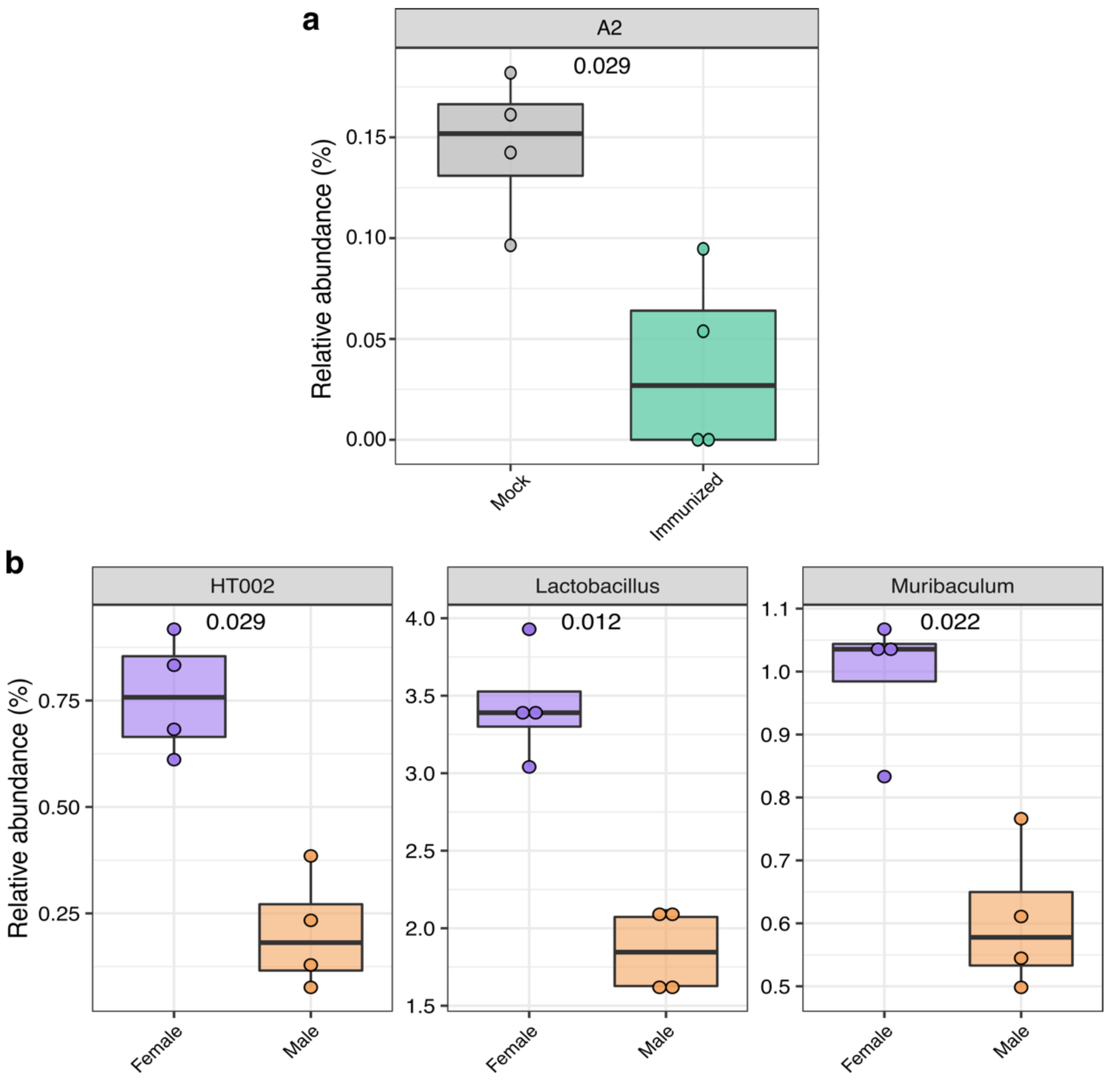

Supplementary Figure 5. Differentially abundant taxa at the baseline. Boxplot comparing pooled fecal microbiome samples by (**a**) experimental groups and (**b**) sex before vaccination (w0, after FMT). Median values interquartile ranges and individual values are shown in boxplots. No statistical significance was found after multiple testing correction (Benjamini-Hochberg False Discovery Rate).

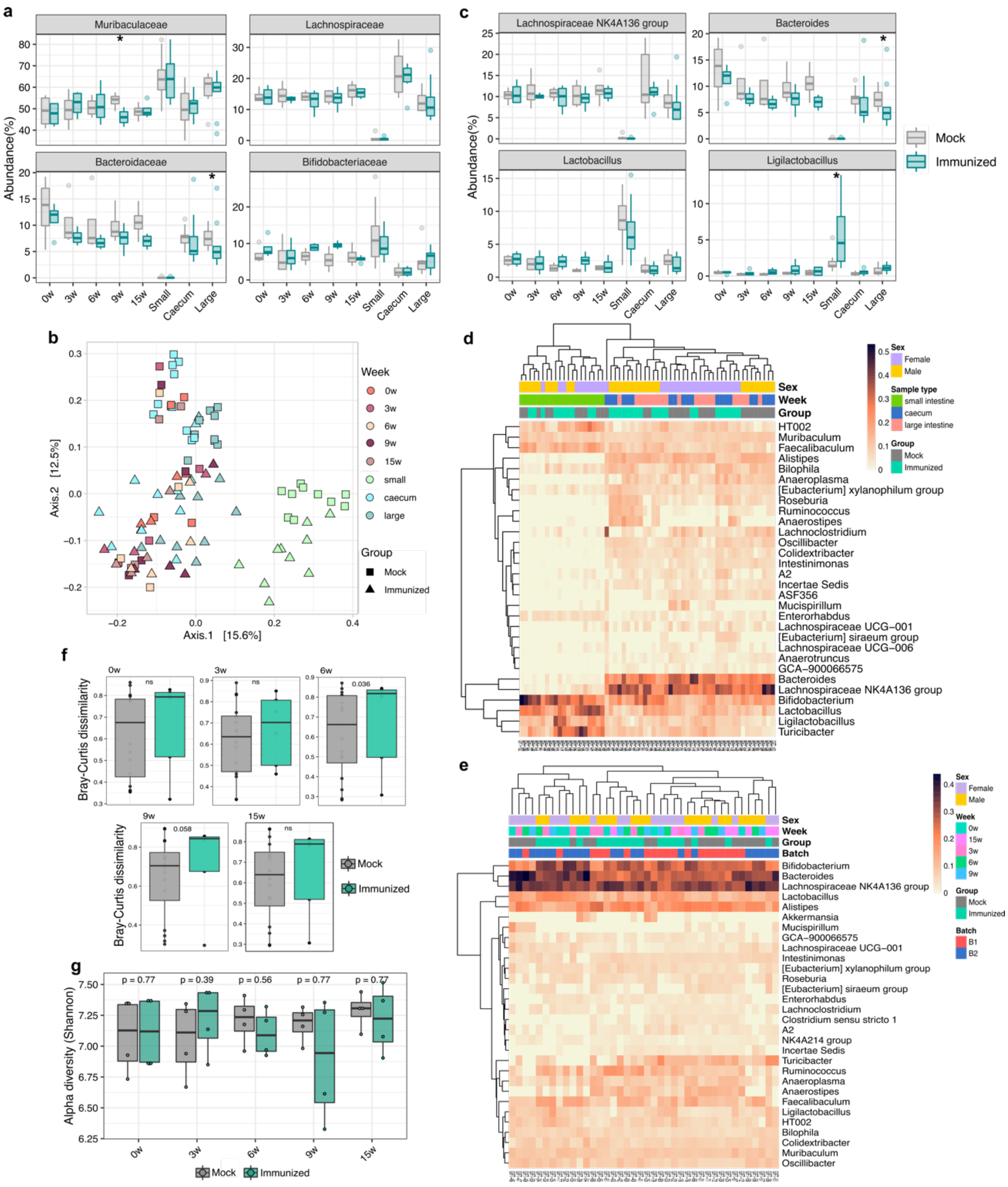

Supplementary Figure 6. Microbial community structure over the study. Longitudinal variations of most abundant bacterial (**a**) families and (**c**) genera. (**b**) PCoA based on Bray-Curtis distances of microbiota profiles (ASV level) from baseline to last time point of the study. Time point and experimental groups are differentiated by colors and shapes, respectively. Hierarchical clustering heatmap of bacterial genera (top 30 most abundant genera are shown) in (**d**) fecal samples from the intestinal content (small intestine, caecum and large intestine) at last point of the study and **(e)** pooled feces from cages. Each column represents data from a single mouse. **(f)**

Boxplots comparing Bray-Curtis dissimilarity index between Mock and Immunized groups. **(g)** Boxplots comparing alpha diversity (Shannon index) pooled fecal samples from Mock and Immunized mice. Median values and interquartile ranges are shown in boxplots. *p*-values from Wilcoxon signed rank test are reported.

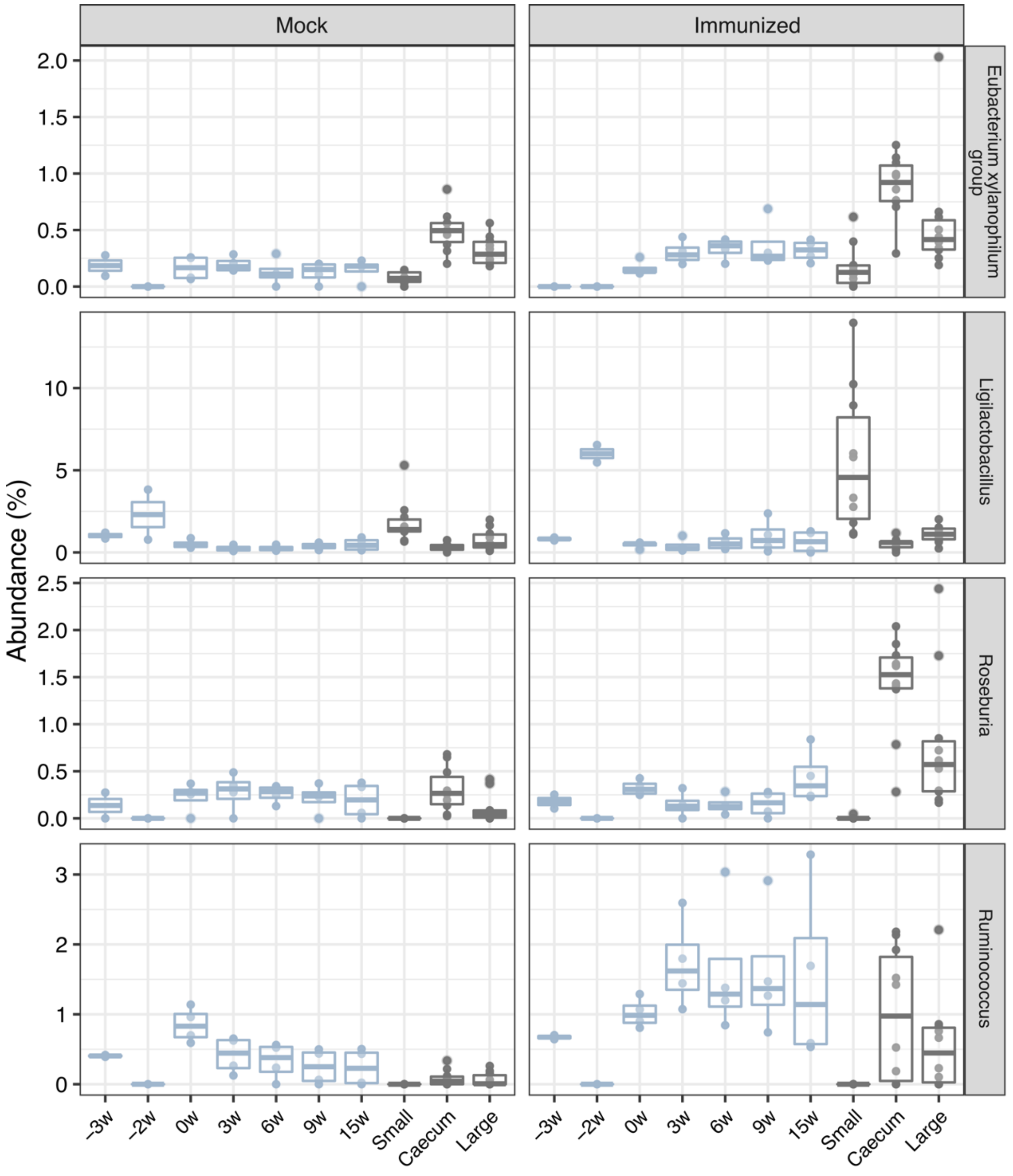

Supplementary Figure 7. Longitudinal assessment of HTI vaccine-associated bacterial genera. Boxplots showing the abundance of genera enriched in the Immunized group over time, identified by LEfSe (LDA>2.0). Evaluation of abundances from mice arrival to last time point of the study in both Mock and Immunized groups is reported. Fecal samples obtained from cages and each intestinal section are represented in light blue and gray, respectively. Median values and interquartile ranges are shown.

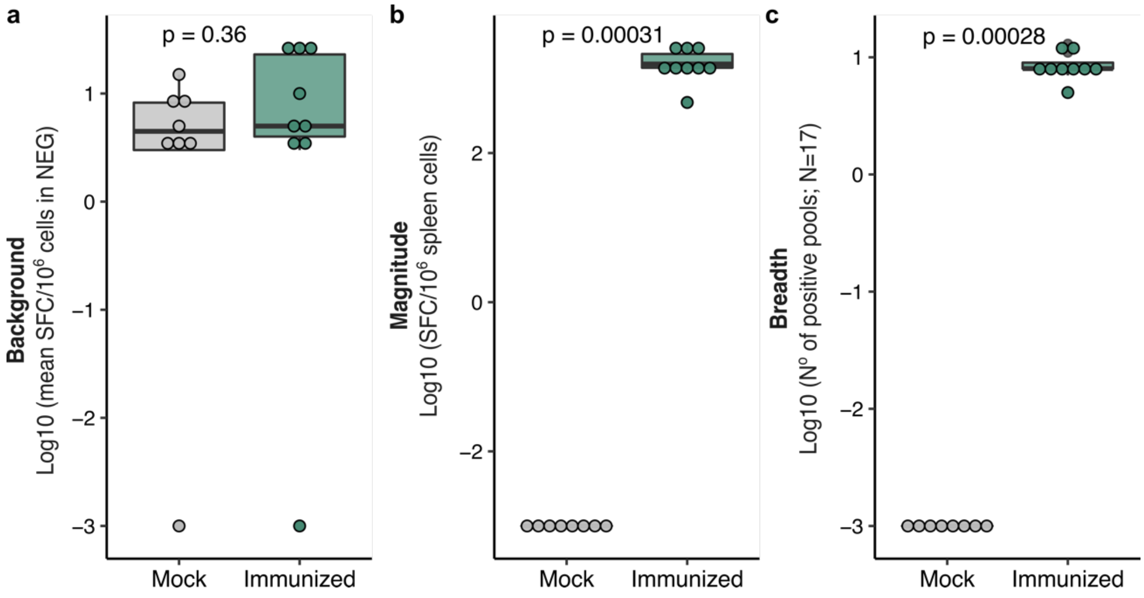

Supplementary Figure 8. Immunogenicity upon HTI vaccination in mice. (**a**) Boxplots comparing the background IFN-γ producing splenocytes (mean of non-stimulated triplicate wells) between PBS- and HTI-vaccinated mice. Magnitude **(b)** and breadth **(c)** of induced HTI-specific IFN-γ responses in PBS- and HTI-vaccinated mice at last time of the study. Median values, interquartile ranges and individual values are shown.

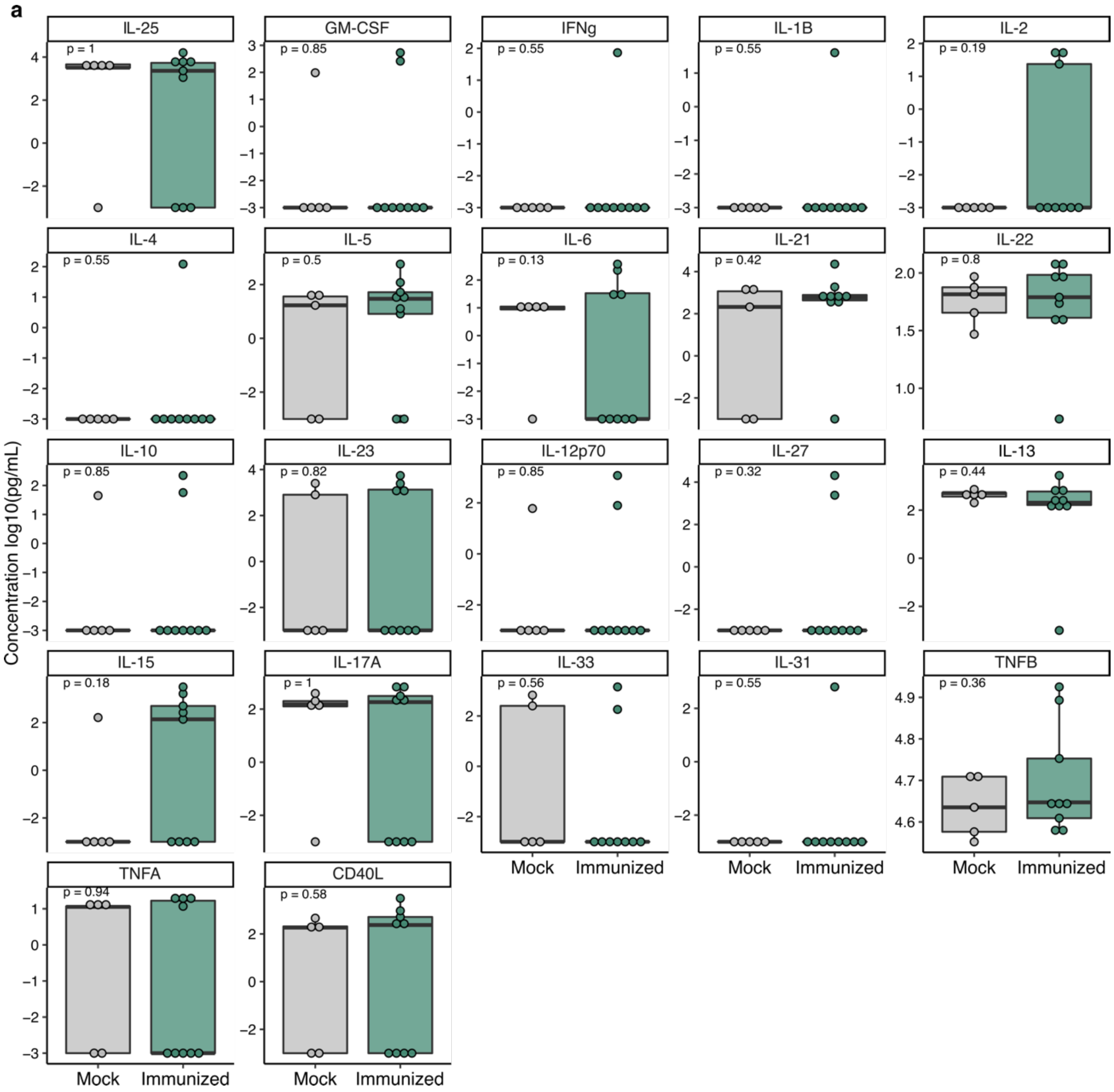
a
Concentration log10(pg/mL)
IL-25
p = 1
GM-CSF
p = 0.85
IFNg
p = 0.55
IL-1B
p = 0.55
IL-2
p = 0.19
IL-4
p = 0.55
IL-5
p = 0.5
IL-6
p = 0.13
IL-21
p = 0.42
IL-22
p = 0.8
IL-10
p = 0.85
IL-23
p = 0.82
IL-12p70
p = 0.85
IL-27
p = 0.32
IL-13
p = 0.44
IL-15
p = 0.18
IL-17A
p = 1
IL-33
p = 0.56
IL-31
p = 0.55
TNFB
p = 0.36
TNFA
p = 0.94
CD40L
p = 0.58
Mock
Immunized

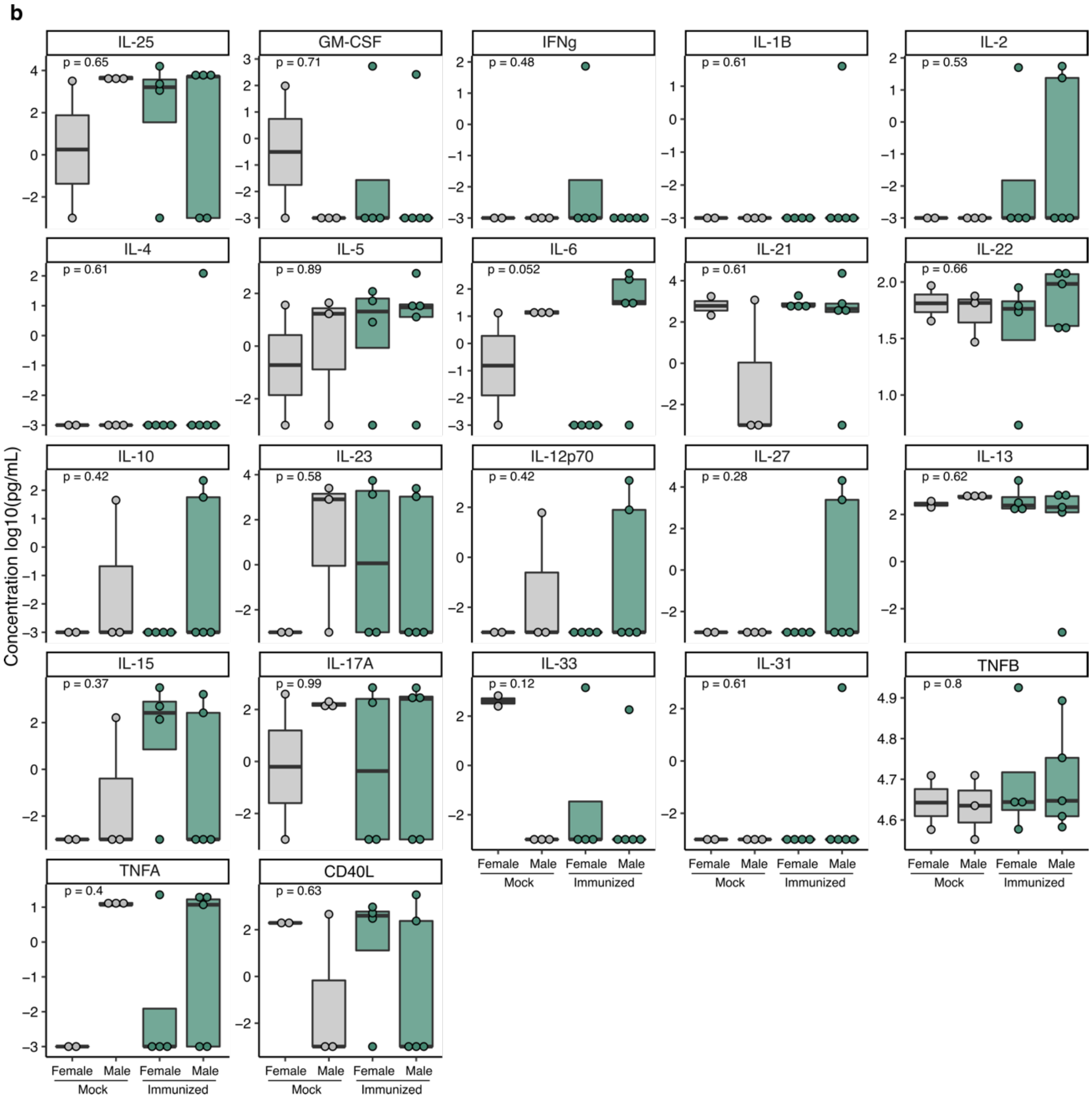

Supplementary Figure 9. Comparison of cytokine levels between Mock and Immunized groups. Boxplots showing serum cytokine levels in (a) Mock and Immunized mice and (b) stratified by sex. Median values, interquartile ranges and individual values are shown.

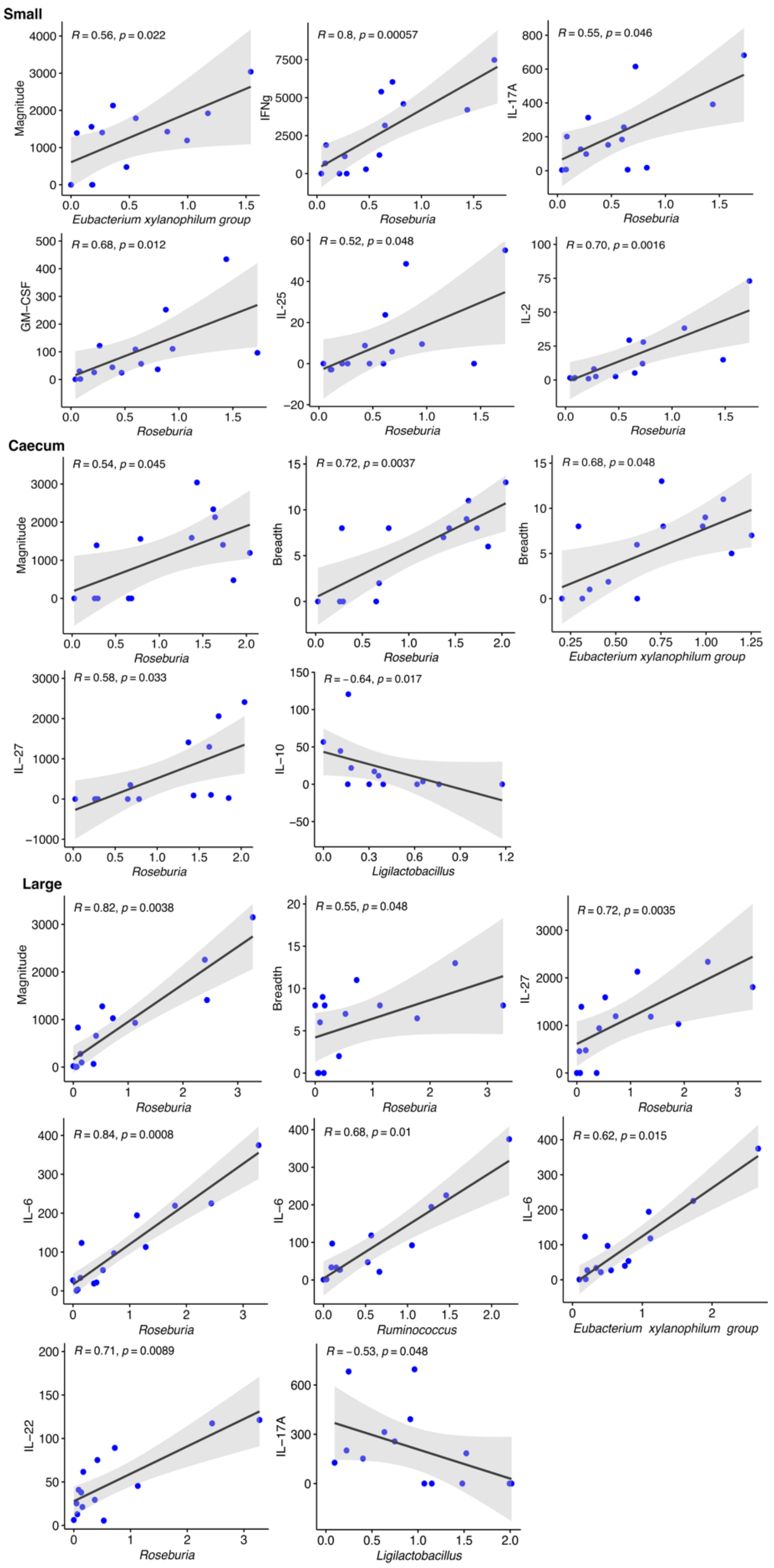

Supplementary Figure 10. Significant correlations between HTI-enriched genera, immunological response and cytokines. Scatterplots showing Spearman's correlation between HTI-enriched bacteria (relative abundance, %), immunological responses (magnitude, SCF/10^6

spleen cells and breadth, N° of positive pools) and serum cytokines (concentration, pg/mL). Only correlations showing significant adjusted p-value are shown. Correlation coefficients and BH-adjusted p-values are indicated on the top of each panel

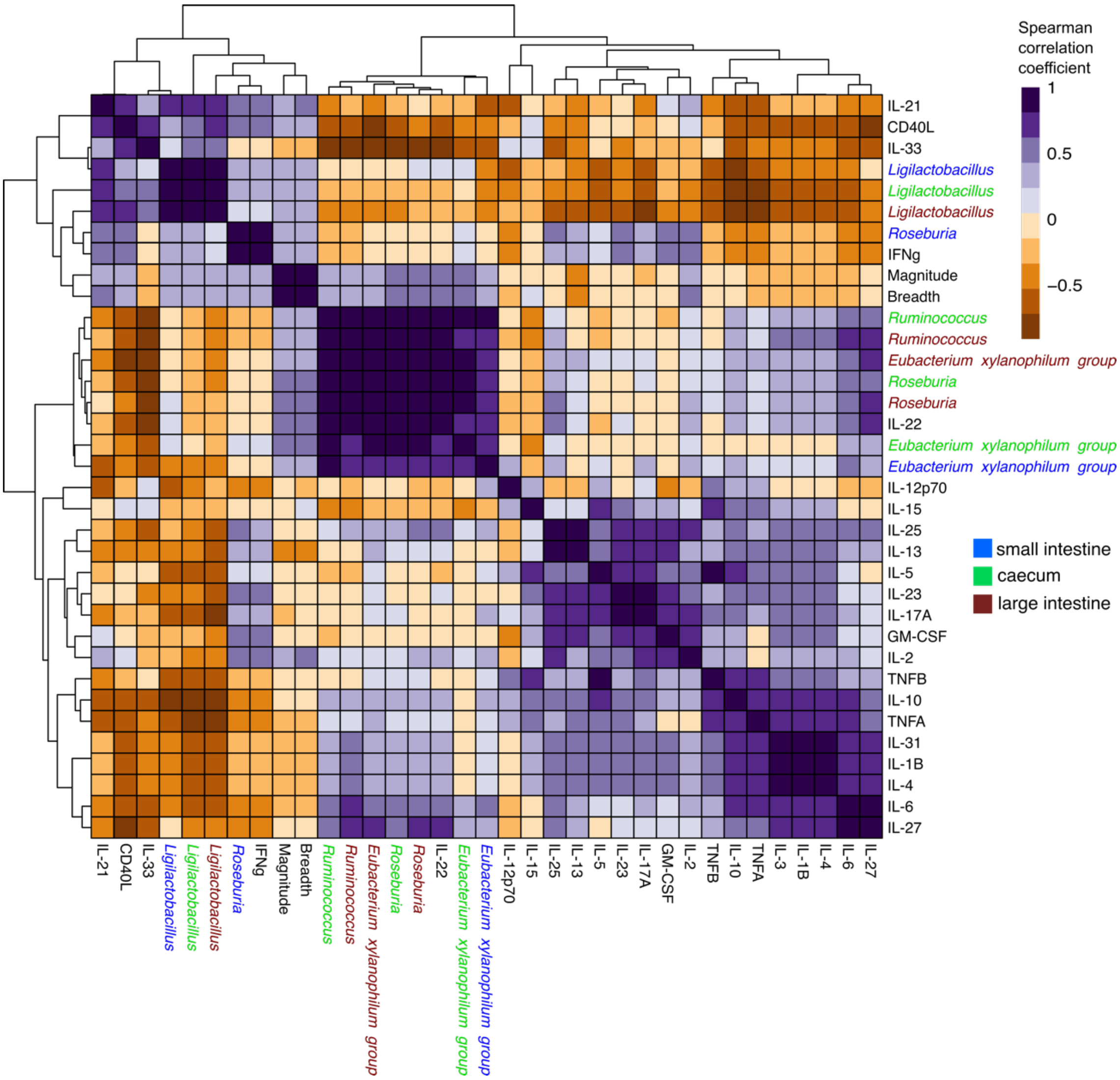

Supplementary Figure 11. Associations between discriminant bacterial genera and immunological responses at last time point of the study. Hierarchical clustering heatmap based on Spearman correlations between bacterial genera (taxa longitudinally enriched in HTI-immunized mice), HTI immunogenicity (magnitude and breadth) and serum cytokine levels. Bacteria from distinct gut sections are differentiated by colors. Correlation coefficients are represented as indicated by the color key.
